# Alectinib in the treatment of ocular metastases of ALK rearranged non small cell lung cancer: Description of 2 case reports

**DOI:** 10.1097/MD.0000000000021004

**Published:** 2020-07-02

**Authors:** Elisa Gozzi, Francesco Angelini, Luigi Rossi, Valentina Leoni, Patrizia Trenta, Giuseppe Cimino, Silverio Tomao

**Affiliations:** a UOC of Oncology, University of Rome “Sapienza,” Aprilia (LT); b Medical Oncology Unit, Regina Apostolorum Hospital, Albano; c Department of Medical Oncology, Sapienza University of Rome, Medical and Surgical Sciences and Biotechnology; d Division of Medical Oncology A, Policlinico Umberto I, Sapienza University of Rome, Rome; e Consorzio Interuniversitario per la Bio-Oncologia (CINBO), Chieti, Italy.

**Keywords:** Alectinib, Alk inhibitors, Alk rearranged NSCLC, choroidal metastasis, ocular metastasis

## Abstract

**Rationale:**

Choroidal metastasis is a rare metastatic location although the most common intraocular neoplasm. In general, choroidal metastases respond favorably to systemic therapy targeted toward the primary neoplasm. In patients with choroidal metastasis of ALK rearranged non small cell lung cancer (NSCLC), targeted therapy using Alk inhibitors gradually replaced radiotherapy as the best treatment. Alectinib is a second-generation ALK inhibitors. Here we describe 2 clinical cases of patients with choroidal metastasis of ALK rearranged NSCLC who received Alectinib as first-line therapy achieving disease control and quality of life improvement.

**Patients concerns:**

In case report 1, 62-year-old man presented with scintillated scotomas at the level of the right eye; in case report 2, 69-year-old man presented with respiratory distress, persistent cough resistant to medical therapy, pain, and blurred vision.

**Diagnoses:**

In case report 1, fundus and ultrasonographic examination showed circumscribed choroid thickening with dome-like appearance compatible with repetitive lesion. Computed tomographic/y (CT) showed multiple bilateral pulmonary nodular formations and adenocarcinoma of the lung was diagnosed by a transbronchial biopsy.

In case report 2, CT showed a primary lesion of 36 × 27 mm in the middle lobe with bilateral lung metastases and lymphadenopathies. Multiple hepatic metastases and minor suspicious bone repetitions. A liver biopsy made a diagnosis of adenocarcinoma compatible with pulmonary primitiveness. An ocular fluoroangiography evidenced a left choroidal metastasis.

**Interventions:**

Case report 1, 2, medical treatment with Alectinib 1200 mg/day was initiated.

**Outcomes:**

In case report 1, a few days after beginning the treatment, both systemic symptoms like respiratory distress and low vision were palliated. Reassessment by CT confirmed treatment response. In case report 2, clinically, visus disorders had already improved 2 weeks after beginning treatment. CT showed pulmonary, nodal, and hepatic response. Stability of bone metastases occurred after 2 months. In addition, ocular ultrasonography documented the regression of previously reported lesions confirmed treatment response.

**Lessons:**

Alectinib works very well in intracranial metastases and is assumed to be so on the ocular ones as well, with benefit for the patient in quality of life.

## 1 Introduction

Ocular metastasis occurs in <10% of malignancies.^[1]^

Most are attributable to a primitive breast cancer following lung cancer. Most of them are located at the level of the choroid.

Choroidal metastasis is a rare metastatic location, but the most common intraocular neoplasm.^[1]^

The spectrum of presentations varies and is responsible for visual deterioration with consequently poorer quality of life.

At first, the standard treatment of the metastasis to the orbit was represented by radiotherapy.

However, the long-term complications due to radiotherapy represented a constant concern especially in the age of long-surviving patients thanks to the progress achieved by medical therapy such as the use of molecular target drugs.^[2]^

Alectinib is a second-generation ALK inhibitor capable of overcoming resistance mutations of first generation inhibitors such as Crizotinib, with good central nervous system (CNS) penetration and a good safety profile.^[3]^

Currently, thanks to the growing interest in the quality of life, targeted therapy for choroidal metastasis is thus considered to be superior to radiotherapy.

Here we describe two clinical cases of patients with choroidal metastasis of ALK rearranged NSCLC who received Alectinib as first-line therapy.

## 2 Case report I

A 62-year-old man presented with a 1-month history of scintillated scotomas at the level of the right eye.

A fundus examination was performed, followed by ultrasonographic examination.

It was revealed in the right eye, in correspondence with amelanotic lesion appearing in fundus examination, circumscribed choroid thickening with dome-like appearance compatible with repetitive lesion, maximum height of the scleral plane 2.36 mm, and medium-low internal reflexivity.

CT showed multiple lymphadenopathy in the ilomediastinal area, the largest of which is in the 40 mm subcarenal area, in the celiac-mesenteric and para-aortic area. Multiple bilateral pulmonary nodular formations, the largest in the LSS of 14 mm and of the mid-basal segment of the 16 mm LIS adherent to the mediastinal pleura, are of likely repetitive significance. Hypodense area of the hepatic segment (Dmax 8 mm) suspected for repetitive injury; left adrenal formation (Dmax 15 mm) of repetitive significance. Repetitive lytic lesion at the frontal level paramandian left (Dmax 9 mm) and left parietal at the vertex (Dmax 9 mm).

Adenocarcinoma of the lung was diagnosed by a transbronchial biopsy.

He was staged as having T4N3M1c (Stage IVb).

Genetic testing found him negative for epidermal growth factor receptor (EGFR) mutation; negative PDL1 expression. However, immunohistochemical staining for ALK gene rearrangement showed extensive positivity at the primary pulmonary site and florescent in situ hybridization demonstrated 61% positivity.

He started first-line treatment with Alectinib 1200 mg/day.

We observed a response to treatment both clinically and with instrumental revaluation: a few days after beginning the treatment, both systemic symptoms like respiratory distress and low vision were palliated. Re-assessment by CT confirmed treatment response, and new ocular control with ultrasonographic examination showed regression of previously observed lesions.

We have not documented any side effects to the treatment.

At time of writing, patient is continuing treatment with Alectinib with excellent disease control.

The patient has provided informed consent for publication of this case report.

### 2.1 Case report 2

A 69 year old man, following systemic signs and symptoms consistent with a respiratory distress, persistent cough resistant to medical therapy, pain and blurred vision, performed a series of specific checks.

CT showed a primary lesion of 36 × 27 mm in the middle lobe with bilateral lung metastases and lymphadenopathies. Multiple hepatic metastases and minor suspicious bone repetitions. Liver biopsy made a diagnosis of adenocarcinoma compatible with pulmonary primitiveness.

He tested negative for the EGFR mutation, The PDL1 expression was about 95% and immunohistochemical staining for ALK gene rearrangement showed positivity.

**Figure 1:**
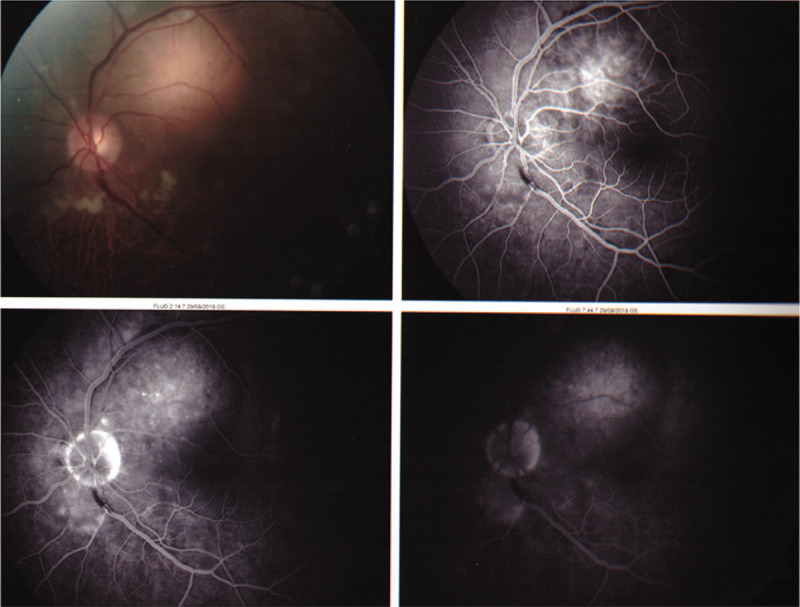
Left choroidal metastasis.

Ocular fluoroangiography evidenced a left choroidal metastasis (Fig. 1). He was staged as having T4N3M1c (Stage IVb). Patient started first-line treatment with Alectinib 1200 mg/day.

We observed a response to treatment both clinically and with instrumental re-assessment. Clinically, visus disorders had already improved 2 weeks after beginning treatment. After two months of beginning treatment, CT showed pulmonary, nodal, and hepatic response, in addiction to stability of bone metastases.

A new revaluation with the ocular ultrasound documented the regression previously reported lesions confirmed treatment response.

We have not documented any side effects to the treatment. At time of writing, patient is continuing treatment with Alectinib with excellent disease control. The patient has provided informed consent for publication of this case report.

## 3 Discussion

The first case of ocular metastases described in literature is attributed to Pears et al in 1872.^[4]^

Ocular metastases occur in 2% to 9% of all malignancies; 69% to 88% of intraocular metastases localize to the choroid.^[5]^

The choroid has the most developed vasculature and highest blood flow of the ocular tissues providing a vascular avenue for tumor emboli to sequester and allows an environment receptive to growth.^[6]^

At the time of ocular diagnosis, 34% have an unknown primary, 47% to 81% are from breast primaries, and 9% to 23% are from lung primaries.^[1]^

Usually, bilateral and multifocal metastases are more often attributable to breast cancer, whereas unilateral and unifocal metastases are found in lung cancer.

Less common primary tumors causing choroidal metastases include carcinoma of the gastrointestinal tract (4%), prostate (2%), kidney (2%–4%), and skin (2%). Rare primary carcinomas metastasizing to choroid include tumors arising from the submandibular gland, thyroid, contralateral choroid, testes, ovaries, urothelial tract, neuroendocrine tumor, and sarcoma.^[5]^

Clinical manifestations associated with choroidal metastases may range from blurred vision (70%–81%) flashes and floaters (5%–12%), and pain (5%–14%) to no symptoms (9%–11%) and lesions may be found on routine ocular examination.^[5]^

At first the standard treatment of the metastasis to the orbit was represented by radiotherapy, (external beam radiotherapy, applying 30 Gy in 10 fractions or 40 Gy in 20 fractions) mainly aimed at palliative intent.^[7]^ However, the long-term complications due to radiotherapy such as cataracts (0.04%), retinopathy, and glaucoma (0.01%) represented a constant concern with significant deterioration in quality of life.^[8]^

In general, before the advent of target molecular therapies, this aspect was not fully considered as the time of development of these complications exceeded the average life expectancy of treated patients.

Choroidal metastases in NSCLC are more commonly found in young patients with negative anamnesis due to habitual smoking, these 2 characteristics, (the young age and the negativity for smoking) are common in the rearranged ALK disease.

However, at present, the relationship between choroidal metastasis mechanisms and rearranged ALK NSCLC is unknown.

In ALK disease target therapies directed against tyrosinase showed better results in survival and in the management of toxicities than chemotherapy.

They are Crizotinib, a first-generation ALK inhibitor,^[9]^ and Alectinib^[10,11]^ and Ceritinib,^[12]^ which are second-generation inhibitors.

Alectinib works very well in intracranial metastases and is therefore assumed to be so on the ocular ones as well, with benefit for the patient in quality of life.^[13,14]^

In a randomized, open-label, phase 3 trial, Peters et al assigned 303 patients with previously untreated, advanced ALK-positive NSCLC to receive either oral Alectinib at a dose of 600 mg twice daily (to be taken with food) or oral Crizotinib at a dose of 250 mg twice daily (to be taken with or without food).

The primary end point was progression-free survival (PFS). Secondary end points were time to CNS progression, objective response rate, and overall survival.^[15]^

The rate of PFS was significantly higher with Alectinib 68.4% than with Crizotinib 48.7% (hazard 0.47; *P* < .001); median PFS with Alectinib was not reached.

The time to CNS progression was significantly longer with Alectinib than with Crizotinib in the intention-to treat population (cause-specific hazard ratio, 0.16; 95% confidence interval (CI), 0.10–0.28; *P* = .001).

A total of 18 patients (12%) in the Alectinib group had an event of CNS progression, as compared with 68 patients (45%) in the Crizotinib group (hazard ratio, *P* < .001).

The cumulative incidence rate of CNS progression, with adjustment for the competing risks of non-CNS progression and death, was consistently lower over time with Alectinib than with Crizotinib, and the 12-month cumulative incidence rate of CNS progression was 9.4% (95% CI, 5.4–14.7) versus 41.4% (95% CI, 33.2–49.4)

Among patients with measurable CNS lesions at baseline, a CNS response occurred in 17 of 21 patients in the Alectinib group (CNS response rate, 81%; 95% CI, 58–95) and in 11 of 22 patients in the Crizotinib group (CNS response rate, 50%; 95% CI, 28–72); 8 patients (38%) in the Alectinib group had a complete CNS response, as compared with 1 patient (5%) in the Crizotinib group.

The median duration of intracranial response was 17.3 months (95% CI, 14.8–not estimable) and 5.5 months (95% CI, 2.1–17.3), respectively.

Among patients with measurable or nonmeasurable CNS lesions at baseline, a CNS response occurred in 38 of 64 patients in the Alectinib group (CNS response rate, 59%; 95% CI, 46–71) and in 15 of 58 patients in the Crizotinib group (CNS response rate, 26%; 95% CI, 15–39); 29 patients (45%) in the Alectinib group had a complete CNS response, as compared with 5 patients (9%) in the Crizotinib group.

A response occurred in 126 patients in the Alectinib group (response rate, 82.9%) and in 114 patients in the Crizotinib group (response rate, 75.5%) (*P* = .09).

As compared with Crizotinib, Alectinib showed superior efficacy in primary treatment of ALK-positive NSCLC.^[15]^

The research of molecular alterations in NSCLC metastatic disease is of fundamental importance for the choice of the best type of treatment: choroidal metastasis responds favorably to systemic therapy targeted toward the primary neoplasm and in patients with choroidal metastasis of ALK rearranged NSCLC, targeted therapy has gradually replaced radiotherapy as the best treatment.

Despite the few cases treated with Alectinib in our center, results are promising reflecting what is described in the literature.

## Author contributions

All authors contributed equally and agree with pubblication of this case report:

Elisa Gozzi was the primary editor and wrote the manuscript. Luigi Rossi conceived the need to describe the case. Francesco Angelini reviewed the literature. Valentina Leoni reviewed the manuscript according to the authors’ instructions. Patrizia Trenta made the English correction. Giuseppe Cimino and Silverio Tomao coordinated the realization of the manuscript. All authors read and approved the final manuscript.
